# Dapagliflozin, sildenafil and their combination in monocrotaline-induced pulmonary arterial hypertension

**DOI:** 10.1186/s12890-022-01939-7

**Published:** 2022-04-12

**Authors:** Yi Tang, Siyuan Tan, Minqi Li, Yijin Tang, Xiaoping Xu, Qinghai Zhang, Qinghua Fu, Mingxiang Tang, Jin He, Yi Zhang, Zhaofen Zheng, Jianqiang Peng, Tengteng Zhu, Wenlin Xie

**Affiliations:** 1 Department of Cardiology, Hunan Provincial People’s Hospital, The First Affiliated Hospital of Hunan Normal University, Clinical Medicine Research Center of Heart Failure of Hunan Province, Hunan Normal University, Changsha, 410005 China; 2 Hunan Provincial People’s Hospital, The First Affiliated Hospital of Hunan Normal University, Changsha, 410005 China; 3 Department of Gastroenterology, Hunan Provincial People’s Hospital, The First Affiliated Hospital of Hunan Normal University, Hunan Normal University, Changsha, 410005 China; 4 Department of Cardiovascular Medicine, The Second Xiangya Hospital, Central South University, Changsha, 410011 China; 5 Department of Pathology, The Seventh Affiliated Hospital of Sun Yat-Sen University, Shenzhen, 518017 China

**Keywords:** Dapagliflozin, Sildenafil, Pulmonary arterial hypertension

## Abstract

**Background:**

Dapagliflozin, a selective inhibitor of sodium-glucose cotransporter 2 (SGLT2), can reduce cardiovascular events and mortality in patients with heart failure. A number of mechanisms have been proposed to explain the beneficial effects of SGLT2 inhibitors. The purpose of this study was to determine whether dapagliflozin can improve pulmonary vascular remodelling and the efficacy of dapagliflozin as an add-on therapy to sildenafil in rats with pulmonary arterial hypertension (PAH).

**Methods:**

A monocrotaline (MCT)-induced PAH rat model was used in our study. MCT-injected rats were randomly divided into four groups and treated for 3 weeks with daily per os treatment with vehicle, dapagliflozin (1 mg/kg/day), sildenafil (25 mg/kg/day), or a combination of dapagliflozin (1 mg/kg/day) and sildenafil (25 mg/kg/day). Haemodynamic measurements, histological analysis, enzyme-linked immunosorbent assay and western blotting analysis were employed to detect the changes in PAH rats after treatments.

**Results:**

Dapagliflozin significantly attenuated MCT-induced increases in right ventricular systolic pressure (RVSP) and right ventricular hypertrophy (RVH) in PAH rats. Dapagliflozin effectively decreased the thickening of pulmonary artery media and decreased the muscularization of pulmonary arterioles in PAH rats. Moreover, dapagliflozin attenuated nucleotide-binding domain-like receptor protein 3 (NLRP3) inflammasome activation in lung tissues and the levels of interleukin-1β (IL-1β) and interleukin-18 (IL-18) in plasma. However, dapagliflozin as an add-on therapy to sildenafil in rats with PAH did not show a more pronounced beneficial effect on right ventricular systolic pressure and pulmonary vascular remodelling in MCT rats than sildenafil alone.

**Conclusions:**

Dapagliflozin reduces right ventricular systolic pressure and pulmonary vascular remodelling in a rat model of PAH. However, combination therapy with dapagliflozin and sildenafil was not more effective than monotherapy with sildenafil in PAH rats.

**Supplementary Information:**

The online version contains supplementary material available at 10.1186/s12890-022-01939-7.

## Background

Pulmonary arterial hypertension (PAH) is a malignant pulmonary vascular disease characterized by increased pulmonary vascular resistance and pulmonary arterial pressure due to pulmonary vascular remodelling, which always results in right heart failure and even death [1]. Previous studies indicated that the prevalence of PAH is approximately 15–60 per million, and the median survival of patients in the absence of targeted drugs is only 2.8 years [2, 3]. Current PAH-targeted agents (phosphodiesterase type-5 (PDE-5) inhibitors, endothelin receptor antagonists, guanylate cyclase stimulators, prostacyclin analogues, and prostacyclin receptor agonists), either by monotherapy or combination therapies, can partially improve patients’ symptoms and haemodynamics by dilating pulmonary vessels. However, the 5-year survival rate is still approximately 50%, even for patients receiving combination therapies [4]. Therefore, it is extremely urgent to explore novel and effective drugs.

The pathogenesis of PAH is still unclear, and a number of mechanisms are involved in the pathophysiological development of PAH [5]. Among them, inflammation plays an important role in the development of pulmonary vascular remodelling. Pulmonary vascular cells can release inflammatory mediators, which can recruit inflammatory cells. Under the coordination of inflammatory mediators, inflammatory cells can promote the release of cytokines, chemokines, and growth factors, which leads to pulmonary vascular remodelling via vascular cell proliferation and collagen deposition [6–8].

Dapagliflozin, a selective inhibitor of sodium-glucose cotransporter 2 (SGLT2), represents a novel therapeutic agent for patients with type 2 diabetes by inhibiting renal reabsorption of glucose. A study further revealed that dapagliflozin contributed to a lower rate of cardiovascular death or hospitalization for patients with heart failure, regardless of the presence or absence of diabetes [9, 10]. A number of mechanisms, such as inflammation, have been proposed to explain the beneficial effects of SGLT2 inhibitors in patients with heart failure [11, 12]. Byrne et al. proved that empagliflozin, another SGLT2 inhibitor, can significantly worsen cardiac dysfunction by inhibiting nucleotide-binding domain-like receptor protein 3 (NLRP3) inflammasome activation in an animal model of heart failure [13].

There is little evidence that SGLT2 inhibitors can improve pulmonary arterial pressure and pulmonary vascular remodelling [14]. Whether dapagliflozin can improve pulmonary vascular remodelling and the potential mechanism are unknown, and its efficacy as an add-on therapy to sildenafil, a targeted agent for PAH, has not yet been studied. The purpose of our study was to determine whether SGLT2 inhibitors can improve pulmonary vascular remodelling by inhibiting NLRP3 inflammasome activation and the potential benefit of combination therapy with dapagliflozin and sildenafil in monocrotaline (MCT)-treated rats, a widely used surrogate model of PAH.

## Materials and methods

### Animal experiments

**Fig. 1:**
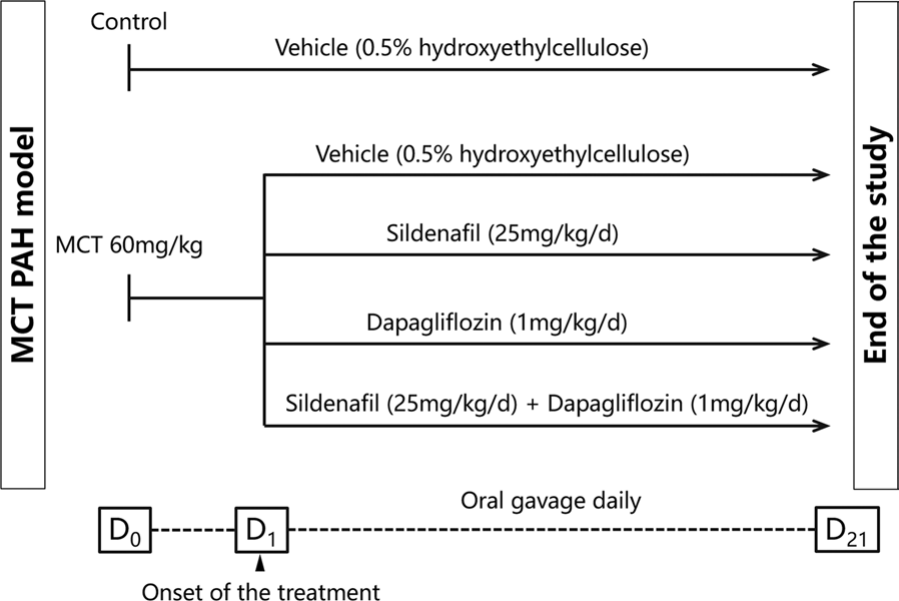
Study design used to test the efficacy of sildenafil, dapagliflozin and sildenafil combined with dapagliflozin in MCT-induced pulmonary arterial hypertension rat model

All animal experiments were performed in accordance with protocols approved by the medical ethics committee of Hunan Provincial People’s Hospital (Approval No. 2020-59). Six-week-old male Sprague–Dawley (SD) rats (160–180 g) were purchased from Hunan SJA Laboratory Animal Company Limted (Hunan, China) and housed in room air (21% O_2_) under a 12-h light and dark cycle. All animals were allowed 1 week to acclimatize to the new environment. MCT (60 mg/kg; Sigma–Aldrich) was dissolved in 1 mol/L HCl, and the pH was adjusted to 7.4 with 1 mol/L NaOH. The PAH model was induced by a single subcutaneous injection of MCT. Control rats received an equal volume of isotonic saline. The body weight of rat in each group was measured every three days. On the day of MCT injection (Day 0), MCT-injected rats were randomly divided into four groups and treated for 3 weeks with daily per os treatment with vehicle [0.5% hydroxyethylcellulose], dapagliflozin (1 mg/kg/day, AstraZeneca Pharmaceutical Co Ltd), sildenafil (25 mg/kg/day, Pfizer Pharmaceuticals Limited), or a combination of dapagliflozin (1 mg/kg/day) and sildenafil (25 mg/kg/day). The experimental protocol is summarized in Fig. 1.

### Haemodynamic measurements

On Day 21, rats were anaesthetized with 2% pentobarbital sodium (40 mg/kg, intraperitoneal) for haemodynamic measurements. A polyethylene-50 (PE50, inner diameter: 0.58 mm, outside diameter: 0.99 mm) catheter was advanced through the right external jugular vein into the right atrium and right ventricle to measure the right ventricular systolic pressure (RVSP). All pressures were recorded by a biological signal acquisition and analysis system (Techman Software Co. LTD, Chendu, China).

### Histological analysis

After completion of right heart catheterization, all animals were euthanized, and both the heart and lungs were dissected and frozen in liquid nitrogen or fixed in 4% paraformaldehyde for further analysis. To evaluate vascular remodelling, the lung was perfused with 4 °C DPBS and perfusion-fixed with 10% formaldehyde solution for 24 h. The whole lung was embedded in paraffin, and sections were made and stained with haematoxylin and eosin (H&E). H&E staining was used to determine the pulmonary artery media thickness (PAMT). At least 10 images of pulmonary arterioles (diameter between 30 and 100 μm) per rat were captured and analysed. The PAMT is defined as the distance between the inner and outer elastic lamina. The vessel external diameter (ED) of the pulmonary arteriole was determined. The relative PAMT (%) was calculated as 100 × 2PAMT/ED.

Fixed lung tissues were stained with anti-alpha-smooth muscle actin (α-SMA, 1:200, Abcam, USA) to detect pulmonary vascular muscularization using immunohistochemistry. Image-Pro Plus version 6.0 software (Media Cybernetics, Inc., Rockville, MD, USA) was used to assess the integrated optical density (IOD) value of the α-SMA IHC section. IODs were obtained as the ratio of sum optical density (OD) to the sum area. Lung tissues were stained with anti-Ki67 (1:200, Abcam, USA) and anti-CD68 (1:100, Abcam, USA) using immunohistochemistry.

The right ventricle (RV) wall was removed from the left ventricle (LV) and the septum (S). The ratio of the weight of RV to the weight of LV + S was calculated to determine the extent of RV hypertrophy.

### Western blotting analysis and plasma IL-1β and IL-18 Assays

Whole blood was collected from the aorta ventralis after haemodynamic measurements. The blood samples were centrifuged at 1000 rpm for 15 minutes, and the plasma was frozen and stored at − 80 °C for future analysis. Concentrations of interleukin-1β (IL-1β) and interleukin-18 (IL-18) in plasma were evaluated using rat IL-1β ELISA Kits (abs530002, absin, Shanghai, China) and IL-18 ELISA Kits (csb-e04610r, cusabio, Wuhan, China) according to the manufacturer’s instructions.

Isolated lung tissues were lysed in RIPA lysis buffer (Beyotime, Shanghai, China) supplemented with 1% protease inhibitor cocktail (Beyotime, Shanghai, China). Protein samples were separated on an SDS–PAGE gel and polyvinylidene fluoride membranes. Next, the membranes were blocked with 5% nonfat milk powder in Tris-buffered saline containing 0.1% Tween 20 for 1 h at room temperature, and then the membranes were probed overnight at 4 °C with the following primary antibodies: rabbit anti-NLRP3 antibody (1:1000, ABclonal, Wuhan, China), rabbit anti-IL-18 antibody (1:1000, Promab, Richmond, USA), rabbit anti-IL-1β antibody (1:1000, Promab, Richmond, USA) and mouse anti-β-actin monoclonal antibody (1:2000, Proteintech, Chicago, USA). Then, the blot was incubated with the appropriate horseradish peroxidase (HRP)-conjugated rabbit IgG (1:5000, Promega, Wisconsin, USA). The signals were visualized using an enhanced chemiluminescence (ECL) reagent kit (Bio-Rad Laboratories, California, USA) and analysed with a Bio-Rad Gel Doc/Chemi Doc Imaging System and ImageJ software. The blots were cut prior to hybridisation with antibodies during blotting.

### Statistical analyses

Data are expressed as the mean ± SD. Comparisons between groups were performed using independent-samples Student’s t-tests for normally distributed variables and the Mann–Whitney U test for nonnormally distributed variables. A 2-sided *P* < 0.05 was considered statistically significant. Statistical analyses were performed using SPSS version 19.0 (SPSS, Inc.).

## Results

### Body weights and survival rates

During the 21 days growth period, rats in the healthy control group showed a 76% increase in body weight. In contrast, rats in vehicle-treated MCT group, which developed severe PAH, only showed a 37% increase in body weight. MCT rats treated with sildenafil (340.40 ± 17.21 vs. 290.22 ± 18.64 g, *P* < 0.0001), dapagliflozin (329.20 ± 13.01 vs. 290.22 ± 18.64 g, *P* < 0.0001) or the combination of dapagliflozin and sildenafil (349.60 ± 13.44 vs. 290.22 ± 18.64 g, *P* < 0.0001) gained much more weight than rats in vehicle-treated MCT group. The combination treatment of dapagliflozin and sildenafil did not significantly improve the body weight of rats compared with sildenafil monotherapy (340.40 ± 17.21 vs. 349.60 ± 13.44 g, *P* = 0.199) (Additional file 1: Figure S1).

In the vehicle-treated MCT group, one rat died within 21 days after MCT treatment, and all rats in the other groups survived within 21 days. Therefore, the 21-day survival rates in the vehicle, sildenafil, dapagliflozin and sildenafil combined with dapagliflozin groups were 90%, 100%, 100%, and 100%, respectively.

### Dapagliflozin attenuated RVSP and right ventricular hypertrophy

**Fig. 2:**
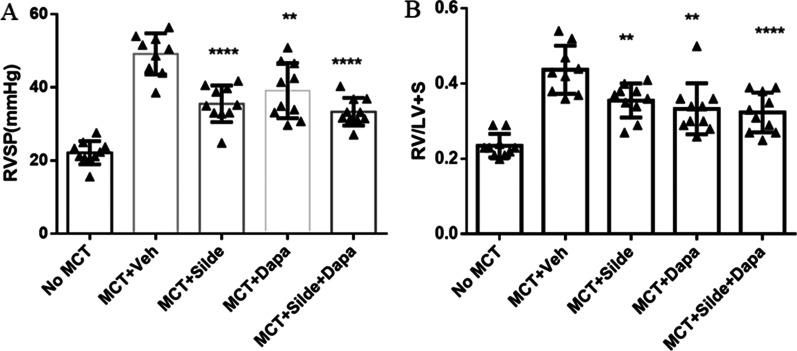
Treatment with dapagliflozin attenuated right ventricular systolic pressure (RVSP) (**A**) and right ventricular hypertrophy (RVH) (**B**) in MCT-injected rats (n = 9–10). Values are means ± SD. Comparisons were made by the Student’s t-test. Dapa, dapagliflozin; MCT, Monocrotaline; Veh, vehicle; Silde, sildenafil. *p < 0.05, **p < 0.01, ***p < 0.001, ****p < 0.0001 versus MCT rats treated with vehicle

The RVSP of SD rats in the vehicle-treated MCT group was significantly increased compared with that of SD rats in the healthy control group (49.14 ± 5.63 vs. 22.14 ± 3.19 mmHg, *P* < 0.0001). The increased RVSP was significantly mitigated by sildenafil (35.52 ± 5.02 vs. 49.14 ± 5.63 mmHg, *P* < 0.0001). Dapagliflozin was also effective in attenuating elevated RVSP (39.11 ± 7.56 vs. 49.14 ± 5.63 mmHg, *P* < 0.005). The combination treatment of dapagliflozin and sildenafil did not significantly further improve RVSP compared with sildenafil monotherapy (33.35 ± 3.76 vs. 35.52 ± 5.02 mmHg, *P* = 0.29) (Fig. 2A).

RVH, as measured by the RV/LV + S ratio, of SD rats in the vehicle-treated MCT group was significantly increased compared with that of SD rats in the healthy control group (0.44 ± 0.06 vs. 0.24 ± 0.03, *P* < 0.0001). Treatment with sildenafil or dapagliflozin alone prevented an increase in RVH (sildenafil: 0.36 ± 0.04 vs. 0.44 ± 0.06, *P* < 0.004; dapagliflozin: 0.33 ± 0.07 vs. 0.44 ± 0.06, *P* < 0.001). The combination treatment with sildenafil and dapagliflozin did not significantly further improve RVH compared with sildenafil monotherapy (0.32 ± 0.05 vs. 0.36 ± 0.04, *P* = 0.13) (Fig. 2B).

### Dapagliflozin attenuated pulmonary vascular remodelling

**Fig. 3:**
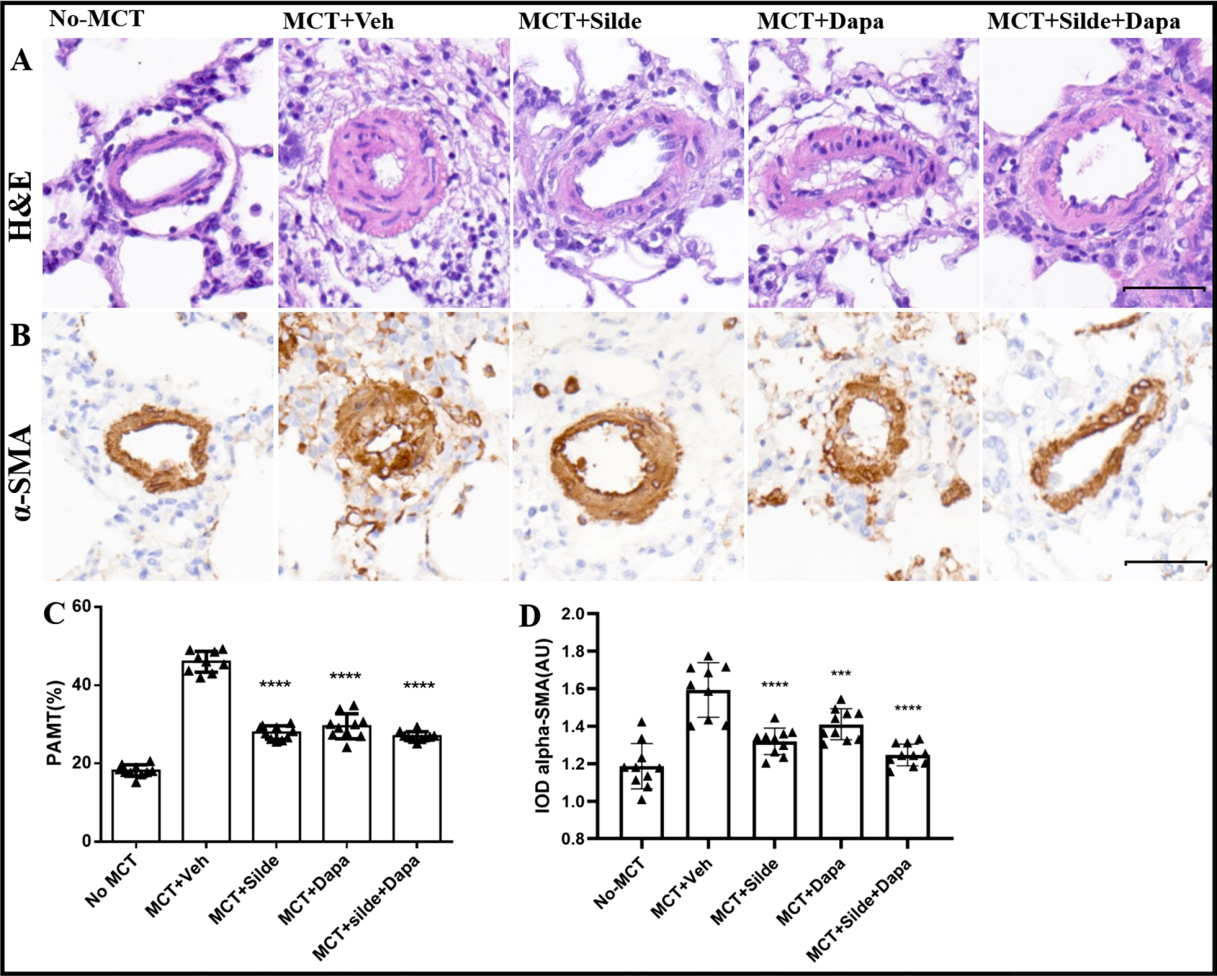
Treatment with dapagliflozin attenuated pulmonary vascular remodelling in MCT-injected rats (n = 9–10). **A** Images of H&E staining for the morphology of pulmonary arteries. **B** Representative images of immunohistochemistry for α-SMA in pulmonary arteries. **C** Quantification of pulmonary artery medial thickness (PAMT). **D** Quantification of α-SMA in pulmonary arteries. Values are means ± SD. Comparisons were made by the Student’s t-test. Dapa, dapagliflozin; MCT, Monocrotaline; Veh, vehicle; Silde, sildenafil. *p < 0.05, **p < 0.01, ***p < 0.001, ****p < 0.0001 versus MCT rats treated with vehicle

**Fig. 4:**
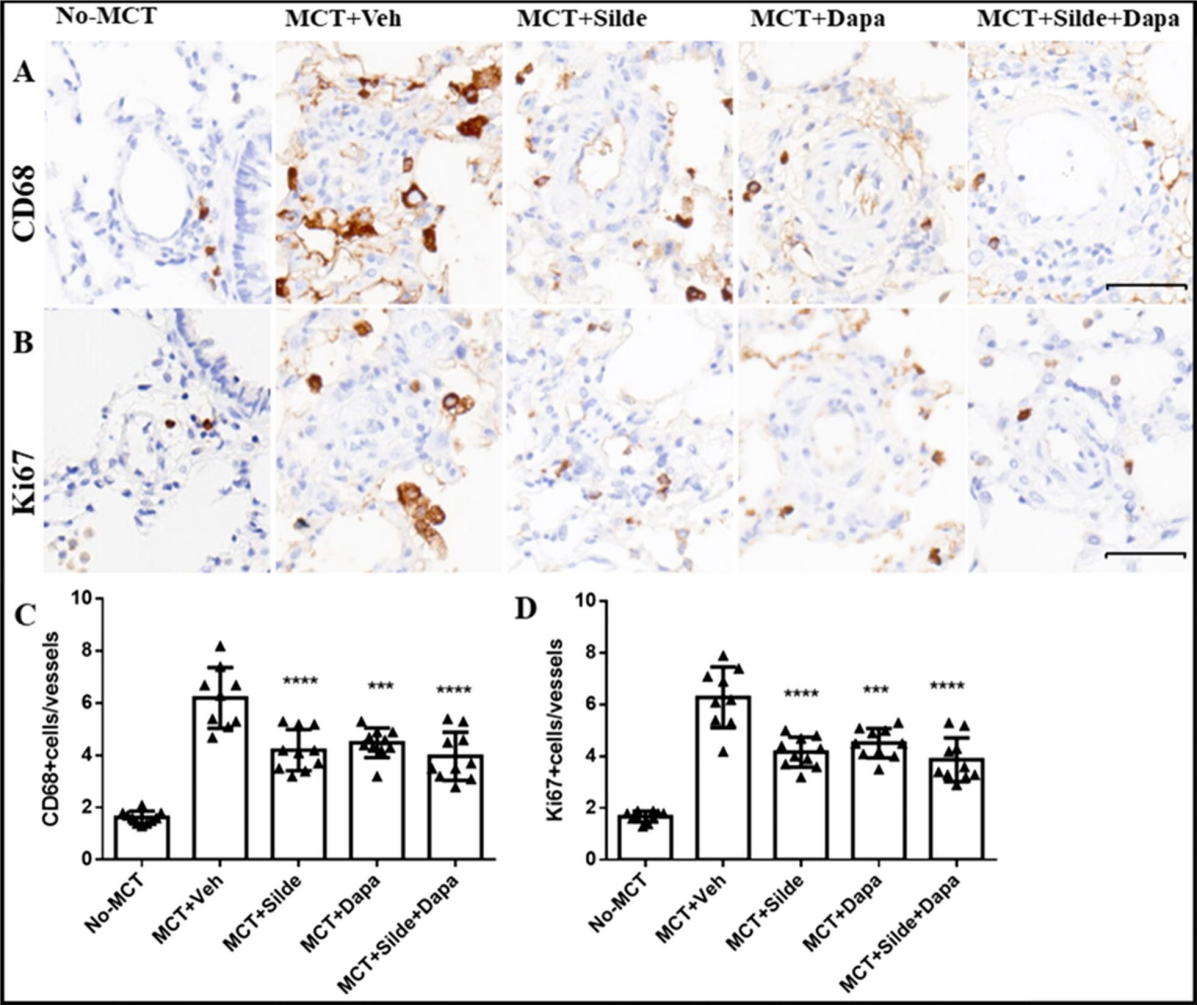
Treatment with dapagliflozin attenuated pulmonary vascular remodelling in MCT-injected rats (n = 9–10). **A** Representative images of immunohistochemistry for CD68 in pulmonary arteries. **B** Representative images of immunohistochemistry for Ki67 in pulmonary arteries. **C** Quantification of CD68-positive cells per pulmonary vessel. **D** Quantification of Ki67-positive cells per pulmonary vessel. Values are means ± SD. Comparisons were made by the Student’s t-test. Dapa, dapagliflozin; MCT, Monocrotaline; Veh, vehicle; Silde, sildenafil. *p < 0.05, **p < 0.01, ***p < 0.001, ****p < 0.0001 versus MCT rats treated with vehicle

Consistent with the increase in RVSP and RVH, vehicle-treated MCT rats also exhibited a significant increase in medial wall thickness compared with rats in the control group (46.00 ± 2.69% vs. 18.18 ± 1.47%, *P* < 0.0001). Dapagliflozin treatment significantly attenuated PA medial wall thickness (29.52 ± 3.20%), which was consistent with the effects of sildenafil (27.91 ± 1.72%). Combination treatment with sildenafil and dapagliflozin further improved PA medial wall thickness, but there was no significant difference between combination treatment and sildenafil monotherapy (26.98 ± 1.16% vs. 27.91 ± 1.72%, *P* = 0.172) (Fig. 3A and C). Dapagliflozin monotherapy treatment also attenuated muscularized pulmonary arteries, as measured by the expression of α-SMA, and there was also no significant difference between combination treatment and sildenafil monotherapy (Fig. 3B and D). The same results were obtained for the numbers of CD68-positive (Fig. 4A and C) and Ki67-positive cells (Fig. 4B and D).

### Dapagliflozin attenuated the expression of NLRP3, interleukin-1β and interleukin-18

**Fig. 5:**
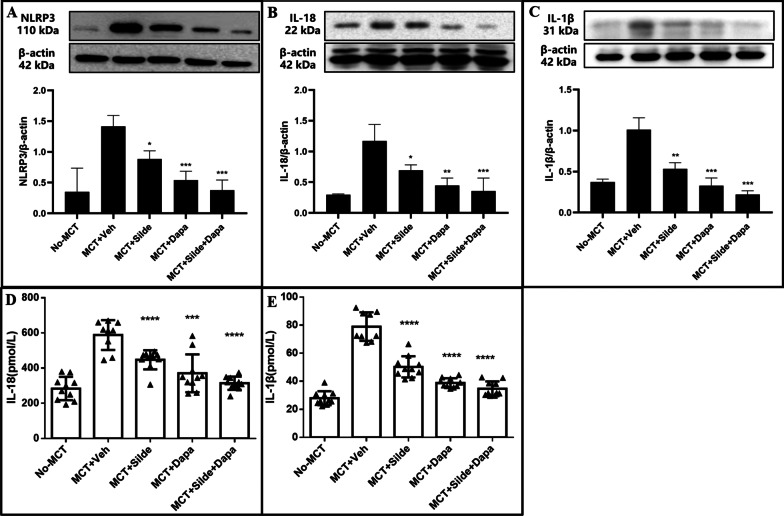
Treatment with dapagliflozin attenuated the expression of NLRP3, IL-1β and IL-18 in MCT-injected rats (n = 3). Western blotting and quantification analysis of NLRP3 (**A**), IL-18 (**B**), IL-1β (**C**) and β-actin (loading control) in lung tissue from different groups. The samples were derived from the same experiment, and the gels/blots were processed in parallel. The gels/blots were corpped, full-length gels/blots are presented in Additional file 2: Figure S2, Additional file 3: Figure S3, and Additional file 4: Figure S4. Quantification of plasma levels of IL-18 (**D**) and IL-1β (**E**). Values are means ± SD. Comparisons were made by the Student’s t-test. Dapa, dapagliflozin; MCT, Monocrotaline; Veh, vehicle; Silde, sildenafil. *p < 0.05, **p < 0.01, ***p < 0.001, ****p < 0.0001 versus MCT rats treated with vehicle

Western blot analysis demonstrated that the expression of the NLRP3 inflammasome in lung tissue was obviously upregulated in vehicle-treated MCT rats compared with that in rats of the control group (1.41 ± 0.18 vs. 0.35 ± 0.38, *P* = 0.012). Compared with MCT rats treated with sildenafil, MCT rats treated with dapagliflozin exhibited a significant decrease in the levels of NLRP3 (0.54 ± 0.15 vs. 0.88 ± 0.13, *P* = 0.039) (Fig. 5A and Additional file 2: Figure S2). The expressions of IL-18 (Fig. 5B and Additional file 3: Figure S3) and IL-1β (Fig. 5C and Additional file 4: Figure S4) in lung tissue were significantly higher in vehicle-treated MCT rats than in rats of the control group (IL-18: 1.17 ± 0.27 vs. 0.29 ± 0.01, *P* = 0.005; IL-1β: 1.01 ± 0.15 vs. 0.37 ± 0.04, *P* = 0.002). Compared with MCT rats treated with sildenafil, MCT rats treated with dapagliflozin exhibited significant decreases of IL-18 (0.45 ± 0.12 vs. 0.70 ± 0.09, *P* = 0.045) and IL-1β (0.33 ± 0.10 vs. 0.53 ± 0.07, *P* = 0.041) in lung tissue.

The plasma concentrations of IL-18 (Fig. 5D) and IL-1β (Fig. 5E) were significantly higher in vehicle-treated MCT rats than in rats of the control group (IL-1β: 78.99 ± 10.25 pmol/L vs. 27.97 ± 4.99 pmol/L, *P* < 0.0001; IL-18: 588.44 ± 84.70 pmol/L vs. 283.69 ± 66.35 pmol/L, *P* < 0.0001). Compared with MCT rats treated with sildenafil, MCT rats treated with dapagliflozin exhibited significant decreases in plasma concentrations of IL-1β (38.91 ± 3.32 pmol/L vs. 50.30 ± 7.61 pmol/L, *P* < 0.0001) and IL-18 (370.71 ± 107.60 pmol/L vs. 447.49 ± 54.45 pmol/L, *P* = 0.027). MCT rats treated with the combination of sildenafil and dapagliflozin exhibited significantly lower levels of IL-1β, IL-18 and NLRP3 than MCT rats treated with sildenafil.

## Discussion

To our knowledge, this is the first study assessing the benefit of dapagliflozin on pulmonary vascular remodeling and as an add-on therapy to sildenafil, a specific PAH treatment in a model of MCT-induced PAH. Our data indicate that dapagliflozin attenuates right ventricular systolic pressure, right ventricular hypertrophy, and pulmonary vascular remodeling and decreases NLRP3 inflammasome activation in MCT-induced PAH rats. However, we found that dual therapy did not show a more pronounced beneficial effect on right ventricular systolic pressure and pulmonary vascular remodeling in MCT rats than sildenafil alone.

Sildenafil, a selective PDE-5 inhibitor, is used in PAH treatment. Sildenafil can relax the pulmonary artery by inhibiting cyclic guanosine monophosphate (cGMP) metabolism. The beneficial effects of sildenafil in PAH have been proven in animal and clinical studies [15–17]. Our study also confirmed that sildenafil could inhibit increases in RVSP and RVH and attenuate pulmonary vascular remodelling. Dapagliflozin, a selective inhibitor of SGLT2, could reduce right ventricle systolic pressure and attenuate pulmonary vascular remodelling in MCT-induced PAH, which was in accord with the results of empagliflozin in MCT-induced PAH [14]. However, the results of our study were different from the results of Huayang Li et al.’s study [15], which may be attributed to 5-week survival rate of monocrotaline (MCT)-induced PAH rats was high and the time of dapagliflozin treatment was longer. To our surprise, the combination therapy of sildenafil and dapagliflozin was not more effective at preventing MCT-induced pulmonary hypertension than sildenafil alone. We speculate the reason may be that sildenafil has already significantly attenuated pulmonary vascular remodelling, endothelial cell dysfunction and chronic inflammation [16, 17], and dapagliflozin cannot further improve inflammation and attenuate pulmonary vascular remodelling. After all, the main mechanism of dapagliflozin is to reduce blood sugar by inhibiting renal reabsorption of glucose. However, the exact mechanism is unknown, further study was needed to identify the reason.

Inflammation plays an important role in the development of pulmonary vascular remodelling [16, 17]. Several studies have demonstrated that the NLRP3 inflammasome and IL-1β play a pivotal role in the pathogenesis of PAH induced by MCT [19–21]. Our study found that the expression of the NLRP3 inflammasome increased and the levels of IL-1β and IL-18 in plasma were higher in vehicle-treated MCT rats than in rats in the control group, which is in accordance with the results of previous studies. Byrne et al. proved that empagliflozin can inhibit NLRP3 inflammasome activation in an animal model of heart failure. Our study also showed that the expression of the NLRP3 inflammasome and the levels of IL-1β and IL-18 significantly decreased in MCT rats treated with dapagliflozin. The results indicated that dapagliflozin may inhibit NLRP3 activation in an animal model of PAH. However, the exact mechanism is unknown, future studies can focus on the effects of dapagliflozin on cell pyroptosis, including detection the change of cell morphological and the protein related to cell pyroptosis, in an MCT-induced PAH model.

## Conclusions

Dapagliflozin attenuated MCT-induced PAH. However, combination therapy with dapagliflozin and sildenafil was not more effective than monotherapy with sildenafil. Further studies with different PAH models or increased treatment durations are required to confirm the beneficial effects of the combination therapy.

## Supplementary Information

**Additional file 1: Figure S1.** The body weights of rats in different groups (n = 9–10). Values are means ± SD. Comparisons were made by the Student’s t-test. Dapa, dapagliflozin; MCT, Monocrotaline; Veh, vehicle; Silde, sildenafil. ***p < 0.001, ****p < 0.0001 versus MCT rats treated with vehicle.

**Additional file 2: Figure S2.** The full-length gels/blots. Western blotting of NLRP3 and β-actin (loading control) in lung tissue from different groups. The right group of image B was deleted for the target protein is not clearly displayed. The samples were derived from the same experiment, and the gels/blots were processed in parallel.

**Additional file 3: Figure S3.** The full-length gels/blots. Western blotting of IL-18 and β-actin (loading control) in lung tissue from different groups. The right group of image B was deleted for this WB group belongs to other experiments. The samples were derived from the same experiment, and the gels/blots were processed in parallel.

**Additional file 4: Figure S4.** The full-length gels/blots. Western blotting of IL-1β and β-actin (loading control) in lung tissue from different groups. The right group of image A was deleted for the internal reference protein has a blank. The right group of image B and left group of image C was deleted for this WB group belongs to other experiments. The samples were derived from the same experiment, and the gels/blots were processed in parallel.

## Data Availability

The datasets used and/or analysed during the current study are available from the corresponding author on reasonable request.

## Abbreviations

PAH: Pulmonary arterial hypertension
MCT: Monocrotaline
PDE-5: Phosphodiesterase type-5
RVSP: Right ventricular systolic pressure
RVH: Right ventricular hypertrophy
NLRP3: Nucleotide-binding domain-like receptor protein 3
IL-1β: Interleukin-1β
IL-18: Interleukin-18
PDE-5: Phosphodiesterase type-5
SGLT2: Sodium-glucose cotransporter 2
SD: Sprague–Dawley
PE50: Polyethylene-50
H&E: Hematoxylin and eosin
PAWT: Pulmonary artery media thickness
ED: External diameter
IOD: Integrated optical density
OD: Optical density
RV: Right ventricle
LV: Left ventricle
S: Septum
HRP: Horseradish-peroxidase
ECL: Enhanced chemilu-minescence
a-SMA: Alpha-smooth muscle actin
