# Supplementary figures and images for: Dapagliflozin, sildenafil and their combination in monocrotaline-induced pulmonary arterial hypertension
Source: BMC Pulm Med. 2022 Apr 12;22:142. doi: 10.1186/s12890-022-01939-7 (PMC9006601; doi:10.1186/s12890-022-01939-7)

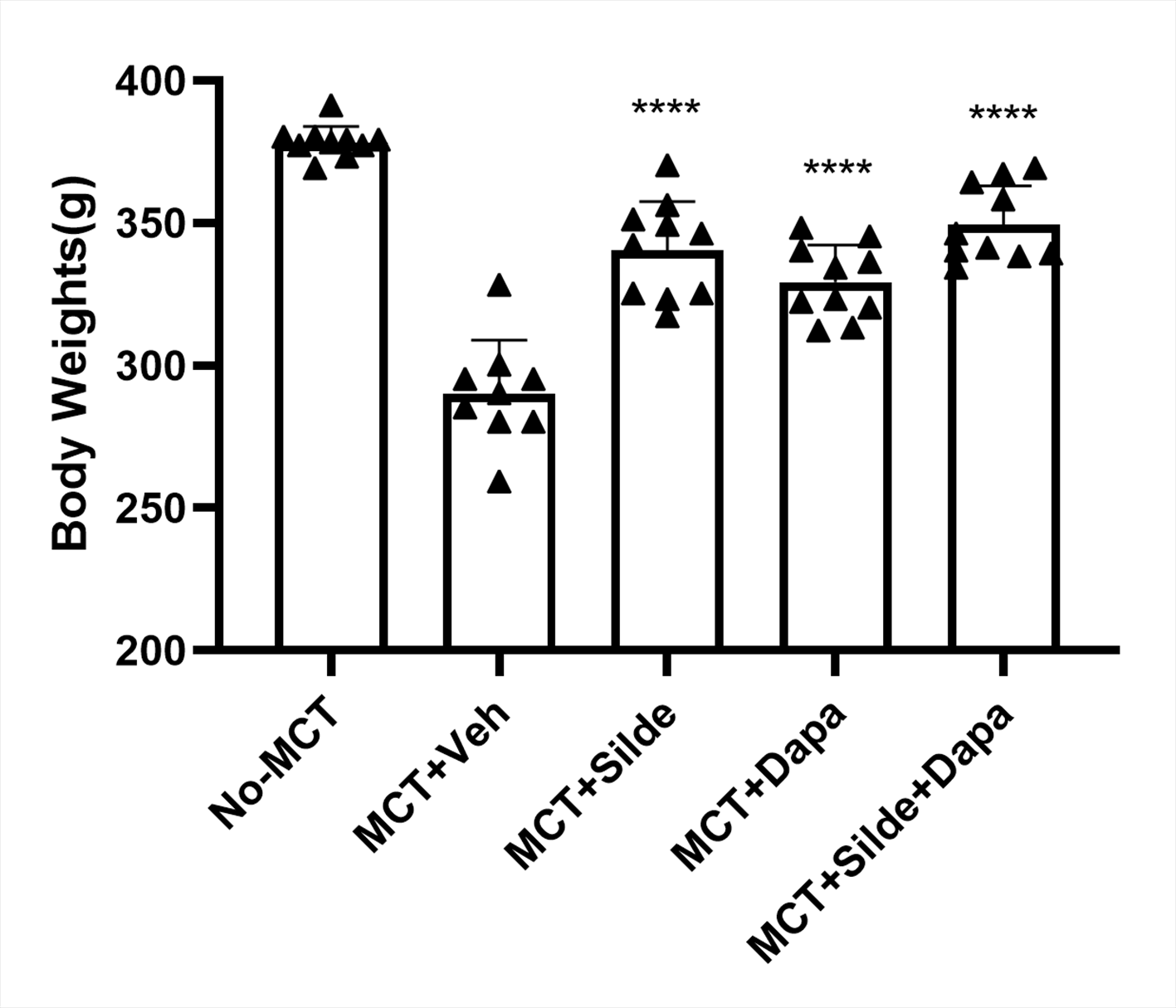

Supplement: Supplementary file 1 — Additional file 1: Figure S1. The body weights of rats in different groups (n = 9–10). Values are means ± SD. Comparisons were made by the Student’s t-test. Dapa, dapagliflozin; MCT, Monocrotaline; Veh, vehicle; Silde, sildenafil. ***p < 0.001, ****p < 0.0001 versus MCT rats treated with vehicle. [file 12890_2022_1939_MOESM1_ESM.tif]

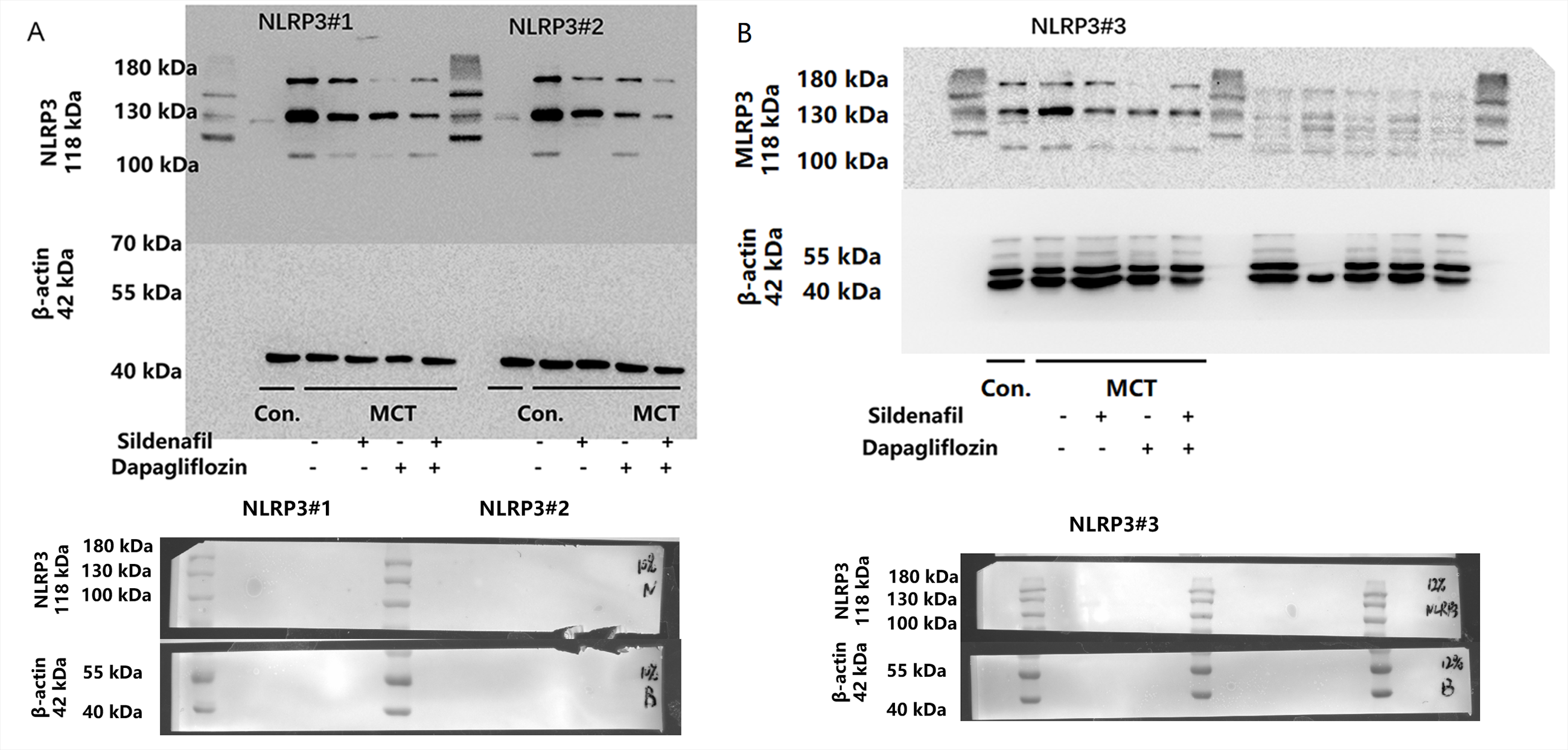

Supplement: Supplementary file 2 — Additional file 2: Figure S2. The full-length gels/blots. Western blotting of NLRP3 and β-actin (loading control) in lung tissue from different groups. The right group of image B was deleted for the target protein is not clearly displayed. The samples were derived from the same experiment, and the gels/blots were processed in parallel. [file 12890_2022_1939_MOESM2_ESM.tif]

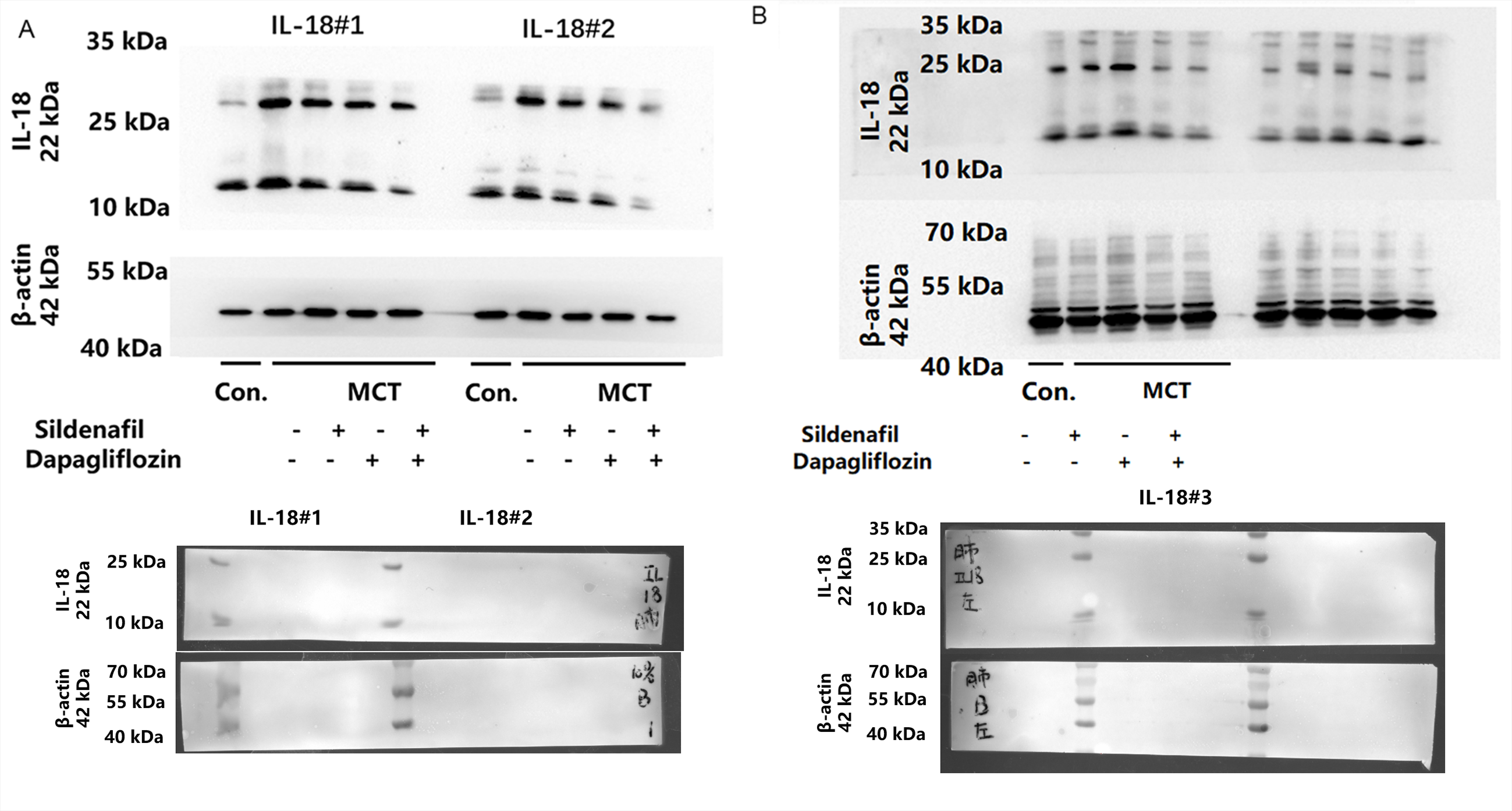

Supplement: Supplementary file 3 — Additional file 3: Figure S3. The full-length gels/blots. Western blotting of IL-18 and β-actin (loading control) in lung tissue from different groups. The right group of image B was deleted for this WB group belongs to other experiments. The samples were derived from the same experiment, and the gels/blots were processed in parallel. [file 12890_2022_1939_MOESM3_ESM.tif]

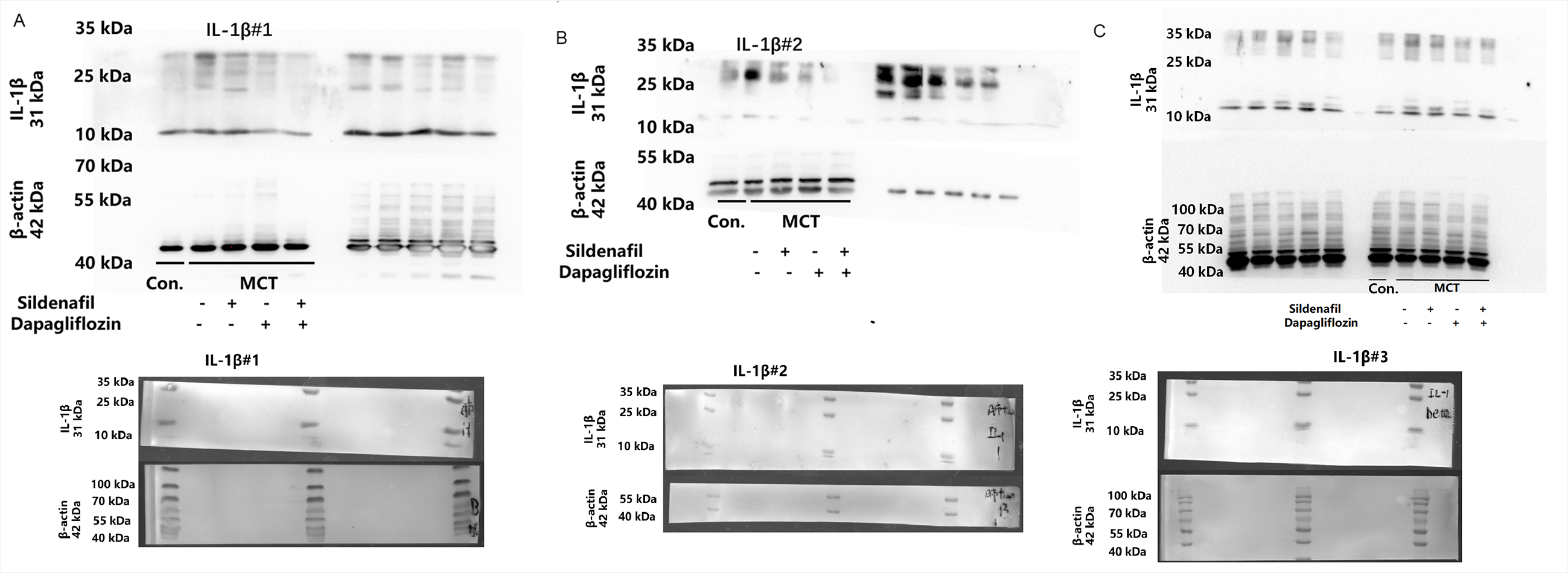

Supplement: Supplementary file 4 — Additional file 4: Figure S4. The full-length gels/blots. Western blotting of IL-1β and β-actin (loading control) in lung tissue from different groups. The right group of image A was deleted for the internal reference protein has a blank. The right group of image B and left group of image C was deleted for this WB group belongs to other experiments. The samples were derived from the same experiment, and the gels/blots were processed in parallel. [file 12890_2022_1939_MOESM4_ESM.tif]
